# Paul Haydon Rogers FRCP FRCPsych: Formerly Consultant Psychiatrist and Medical Director, St Crispin Hospital, Northampton

**DOI:** 10.1192/pb.bp.117.056200

**Published:** 2017-10

**Authors:** Don Williams

**Figure:**
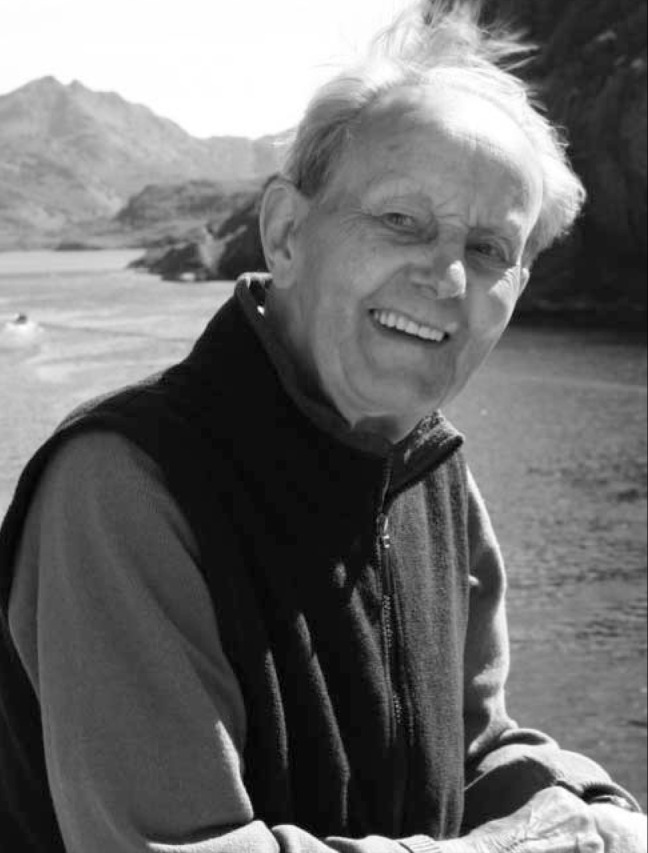


Paul Rogers died on 11 February 2016, aged 96, after a short illness. For the last 10 years of his life he lived independently and happily in a support and care complex in the grounds of St Crispin hospital (now converted into apartments) where he had worked from 1955 to 1979. During his time at St Crispin, where he established community-based services, he was responsible for the introduction of new treatments, particularly the newly developed psychotropic drugs. He developed child guidance clinics in the area and was also involved in the planning of Princess Marina Hospital for people with learning difficulties. After retirement from the National Health Service (NHS) he worked with the Health Advisory Service and as a member of the Mental Health Review Tribunal dealing with difficult issues at Broadmoor and Rampton high-security hospitals. In Northampton, he pioneered the recognition of stress at work and helped to create a local charity to advise employers and provide counselling for employees.

A son of the manse, he was born in Cardiff in 1919 and from an early age wanted to be a doctor. After attending Caterham School and Christ's College Cambridge, he qualified in 1943 at the London Hospital, now The Royal London Hospital. Shortly afterwards, he served as captain in the Royal Army Medical Corps in Normandy, Egypt and Palestine. On demobilisation in 1947 he returned to the London Hospital to pursue his ambition to become a consultant physician. Unfortunately, at this time, there was ferocious competition for promotion because of the high number of doctors returning from the forces after the Second World War. So, instead, he retrained at the Maudsley Hospital to become a psychiatrist. Nevertheless, he remained a physician at heart, adopting a medical approach and placing great emphasis on diagnosis and the use of physical methods of treatment. Although he was insistent that he did not do psychotherapy, he had all the personal qualities of an excellent psychotherapist: empathy, non-possessive warmth, genuineness and a supportive non-judgemental approach.

Paul was a consummate professional dedicated to his work and the NHS, and as a role model he influenced several younger colleagues to take up psychiatry. He had no time for private practice or medical politics. His focus was the care and management of his patients. They loved him and on the rare occasions I took his clinic their disappointment was palpable. Modest, unassuming, understated and unassertive, he combined the qualities of an English gentleman with attributes of Welsh nonconformity and liberalism, reflecting his deep roots in west Wales. Basic Christian values guided his life and he had no regard for social class – a good example of this was his lifelong friendship with his batman, the only link he maintained with his time in the army. The Guardian was his newspaper and he espoused its core values.

He was devoted to his family and only looked for the good in everyone. He supported many charities and was preoccupied with people, who, as he put it, were ‘less fortunate than I am through no fault of their own’. In retirement, activities linked to psychiatry gradually declined and were replaced by his only hobby – gardening. He enjoyed growing food for the house and his garden was always a pleasure to see. Over the years and into advanced old age he enjoyed excellent health; so much so that his close relatives were lulled into thinking that he would always be around and that his support, consideration and wise advice would always be available. He grew old gracefully in every way, retaining his core liberal values to the end.

Marjorie, his wife, and his younger daughter, Helen, predeceased him. He is survived by his sister, daughter and son.

